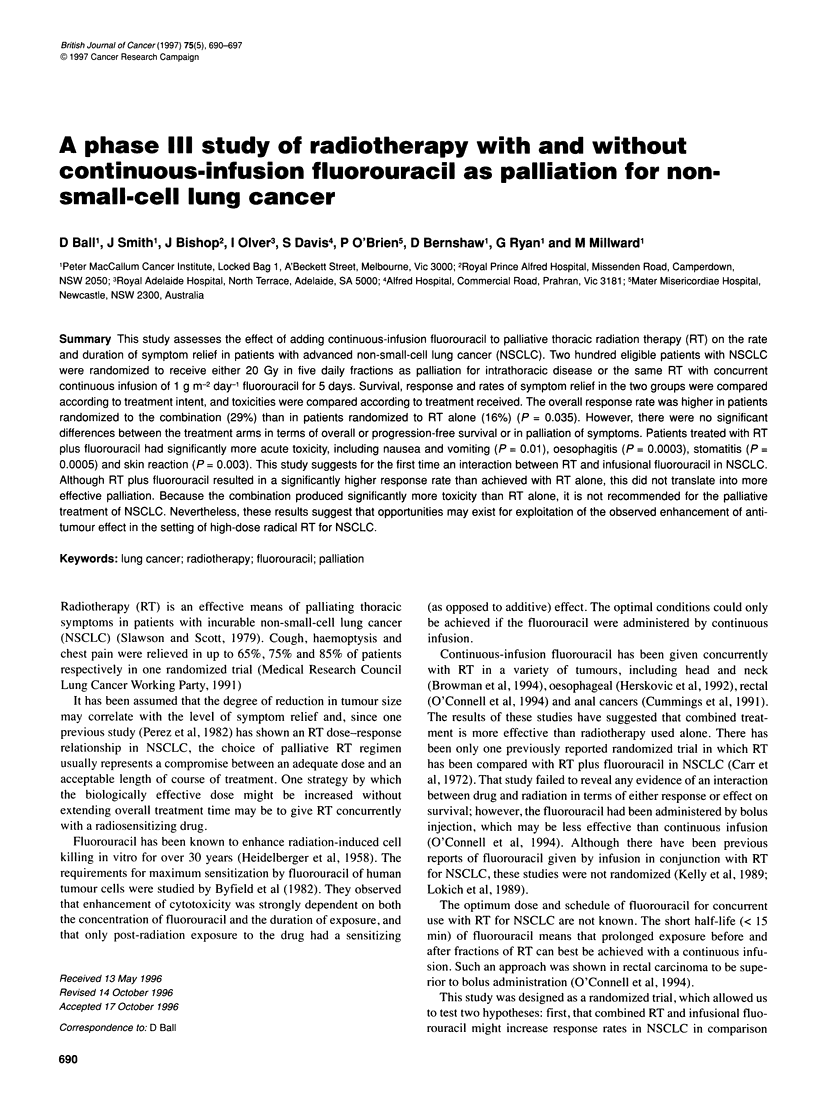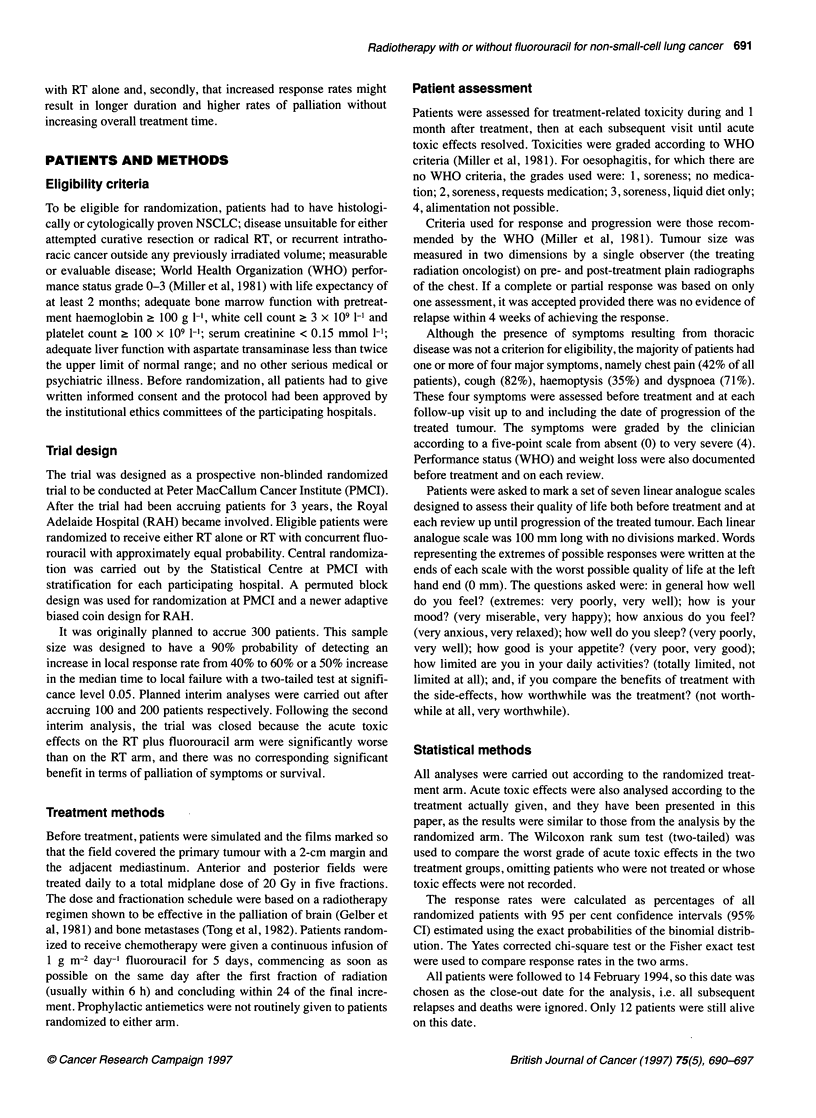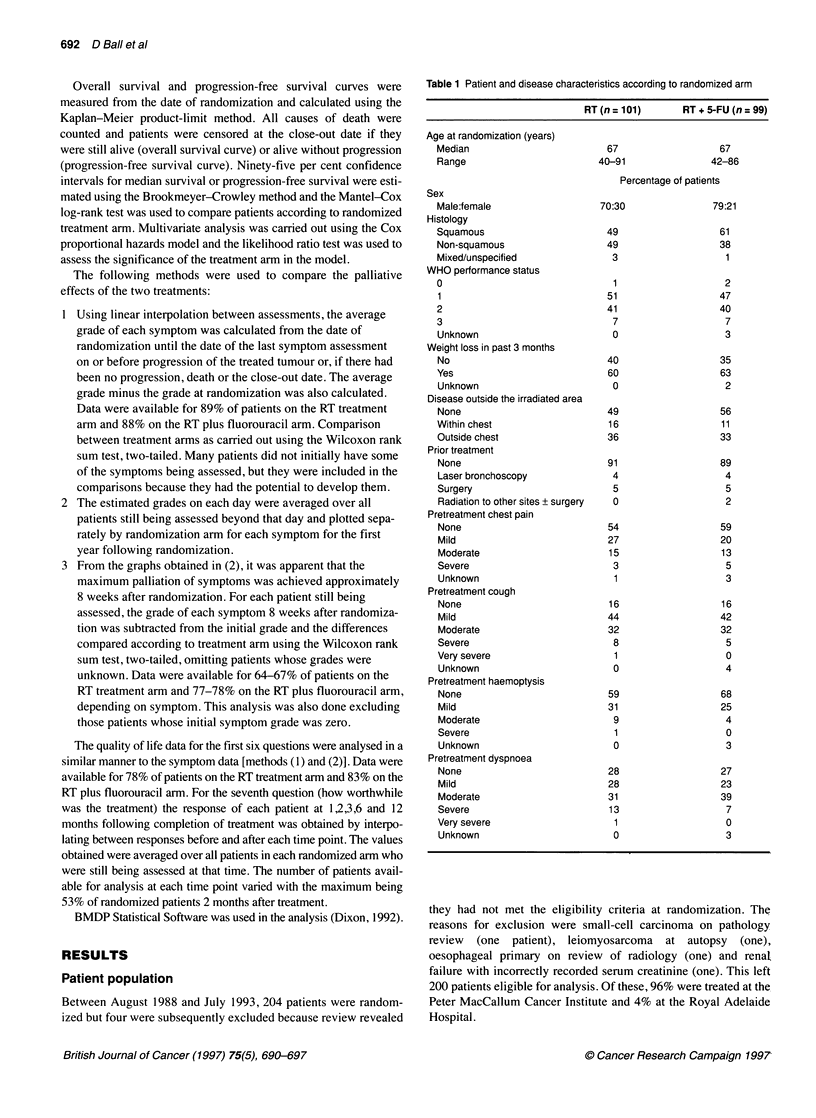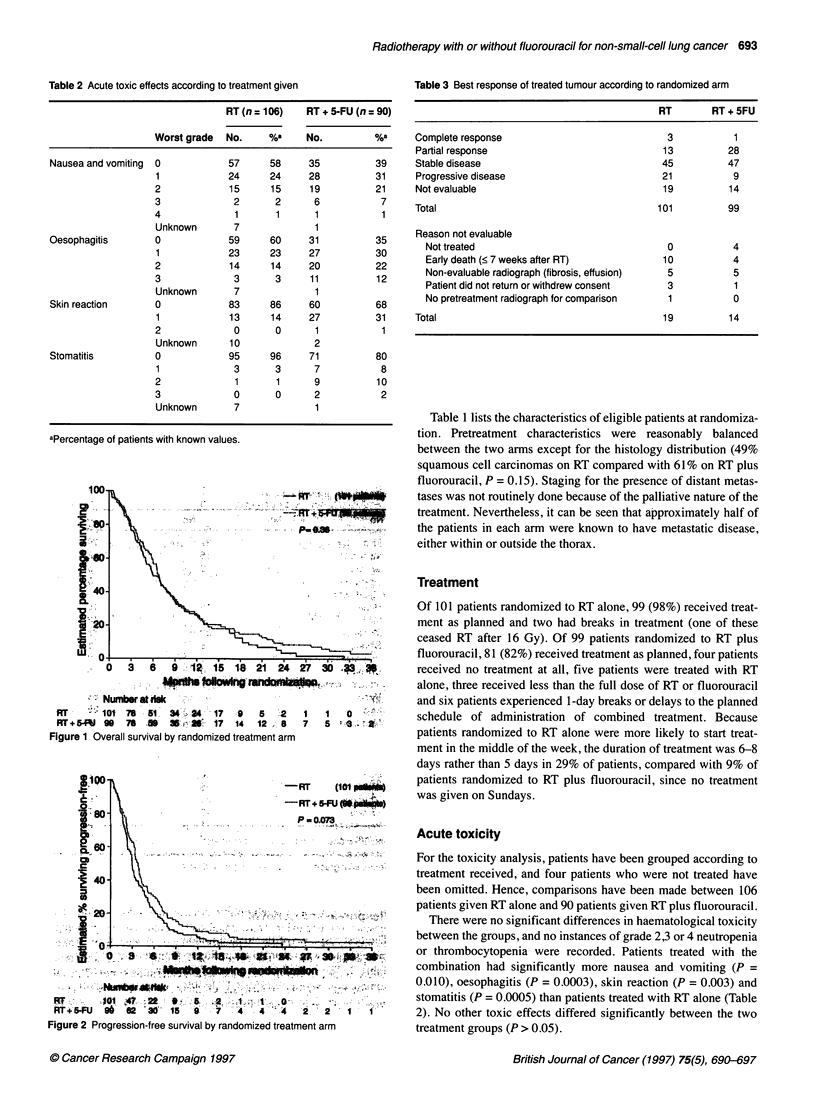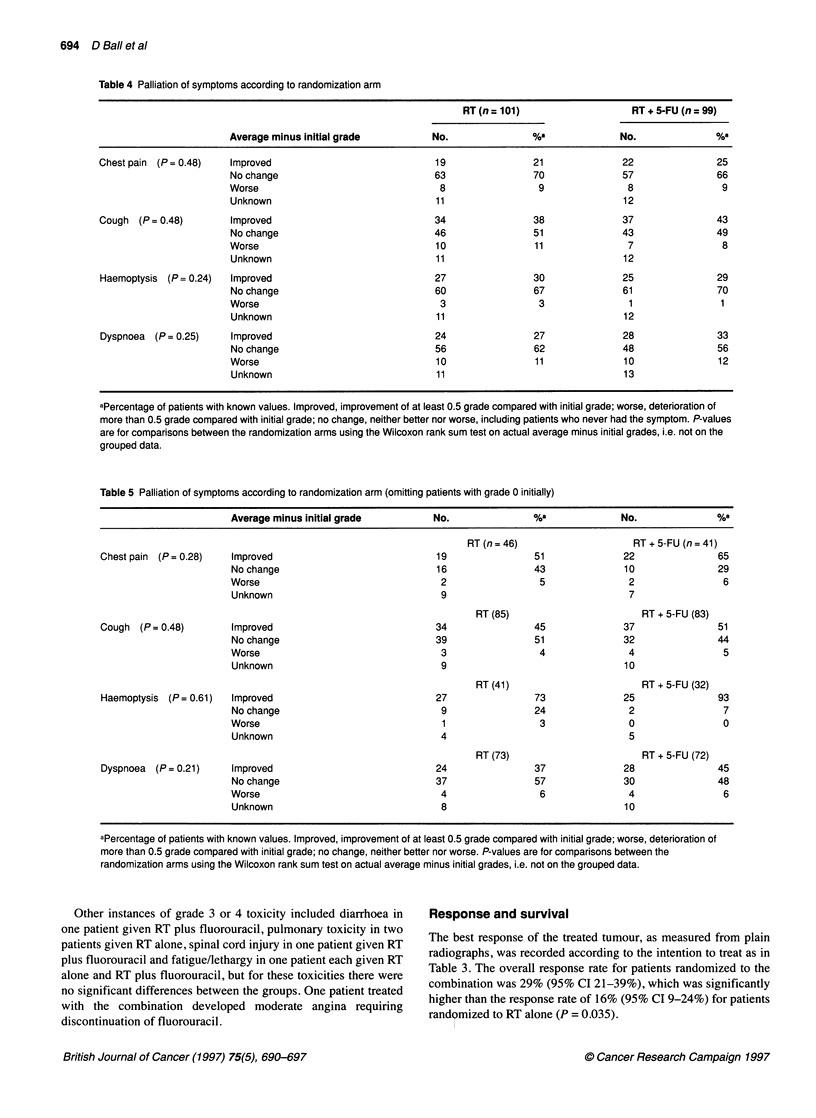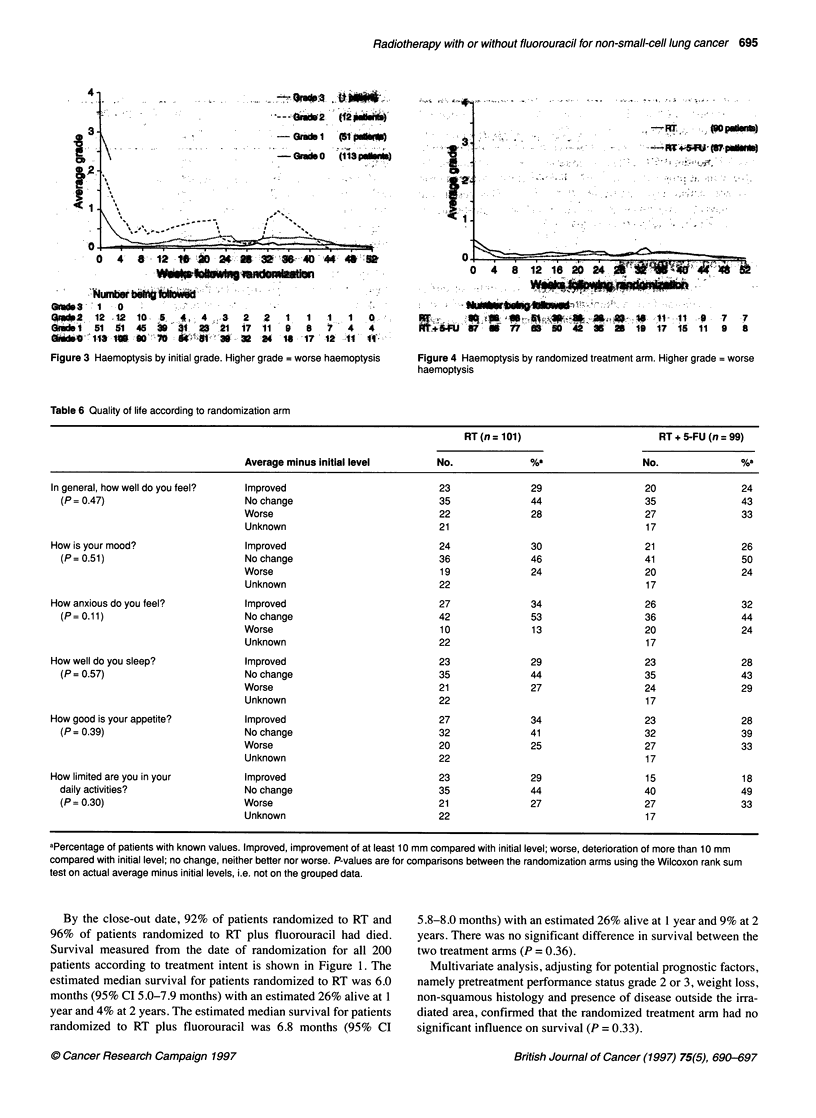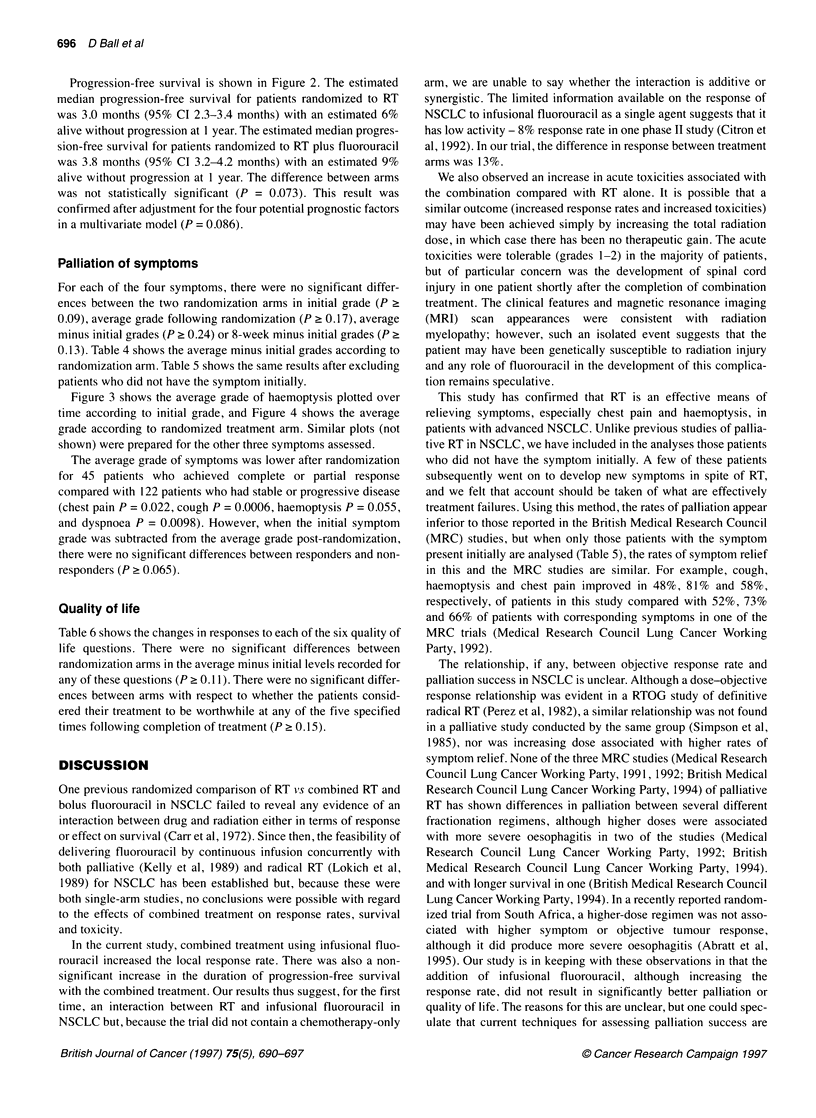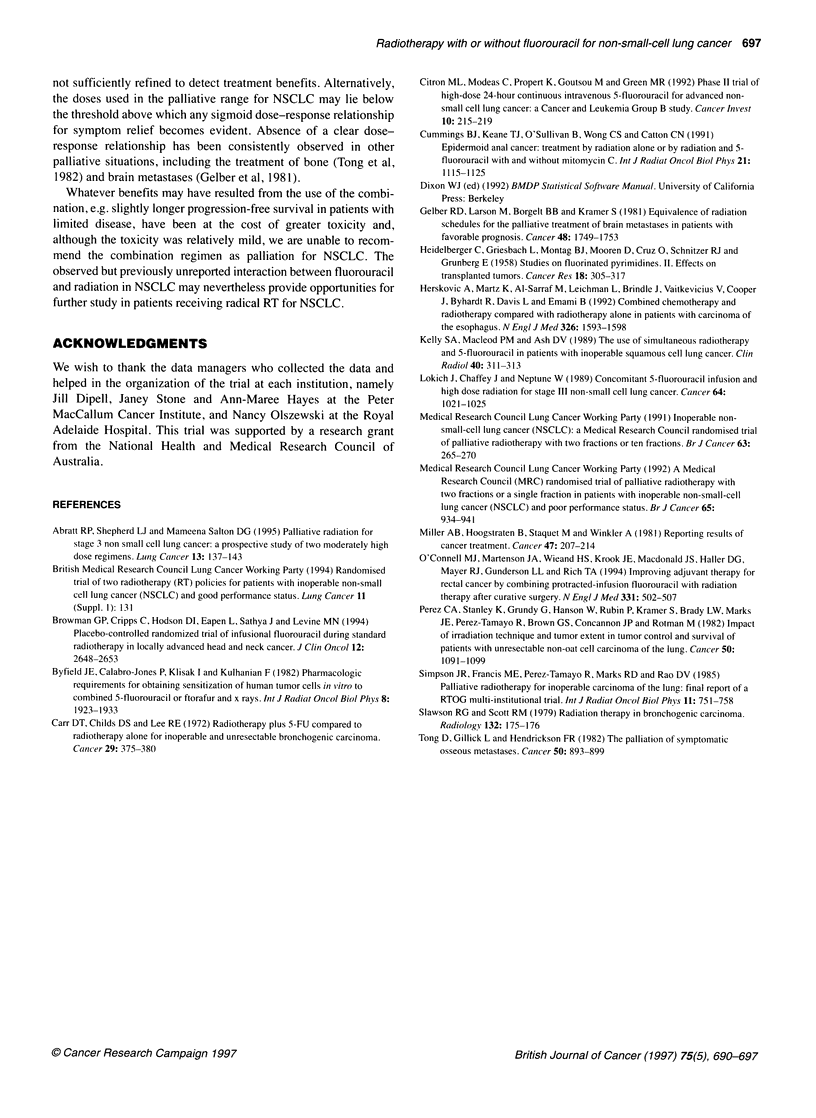# A phase III study of radiotherapy with and without continuous-infusion fluorouracil as palliation for non-small-cell lung cancer.

**DOI:** 10.1038/bjc.1997.123

**Published:** 1997

**Authors:** D. Ball, J. Smith, J. Bishop, I. Olver, S. Davis, P. O'Brien, D. Bernshaw, G. Ryan, M. Millward

**Affiliations:** Peter MacCallum Cancer Institute, Melbourne, Vic, Australia.

## Abstract

This study assesses the effect of adding continuous-infusion fluorouracil to palliative thoracic radiation therapy (RT) on the rate and duration of symptom relief in patients with advanced non-small-cell lung cancer (NSCLC). Two hundred eligible patients with NSCLC were randomized to receive either 20 Gy in five daily fractions as palliation for intrathoracic disease or the same RT with concurrent continuous infusion of 1 g m(-2) day(-1) fluorouracil for 5 days. Survival, response and rates of symptom relief in the two groups were compared according to treatment intent, and toxicities were compared according to treatment received. The overall response rate was higher in patients randomized to the combination (29%) than in patients randomized to RT alone (16%) (P = 0.035). However, there were no significant differences between the treatment arms in terms of overall or progression-free survival or in palliation of symptoms. Patients treated with RT plus fluorouracil had significantly more acute toxicity, including nausea and vomiting (P = 0.01), oesophagitis (P = 0.0003), stomatitis (P = 0.0005) and skin reaction (P = 0.003). This study suggests for the first time an interaction between RT and infusional fluorouracil in NSCLC. Although RT plus fluorouracil resulted in a significantly higher response rate than achieved with RT alone, this did not translate into more effective palliation. Because the combination produced significantly more toxicity than RT alone, it is not recommended for the palliative treatment of NSCLC. Nevertheless, these results suggest that opportunities may exist for exploitation of the observed enhancement of antitumour effect in the setting of high-dose radical RT for NSCLC.


British Journal of Cancer (1997) 75(5), 690–697
© 1997 Cancer Research Campaign

### A phase III study of radiotherapy with and without continuous-infusion fluorouracil as palliation for non-small-cell lung cancer

D Ball[1], J Smith[1], J Bishop[2], I Olver[3], S Davis[4], P O'Brien[5], D Bernshaw[1], G Ryan[1] and M Millward[1]

[1]Peter MacCallum Cancer Institute, Locked Bag 1, A'Beckett Street, Melbourne, Vic 3000; [2]Royal Prince Alfred Hospital, Missenden Road, Camperdown, NSW 2050; [3]Royal Adelaide Hospital, North Terrace, Adelaide, SA 5000; [4]Alfred Hospital, Commercial Road, Prahran, Vic 3181; [5]Mater Misericordiae Hospital, Newcastle, NSW 2300, Australia

**Summary** This study assesses the effect of adding continuous-infusion fluorouracil to palliative thoracic radiation therapy (RT) on the rate and duration of symptom relief in patients with advanced non-small-cell lung cancer (NSCLC). Two hundred eligible patients with NSCLC were randomized to receive either 20 Gy in five daily fractions as palliation for intrathoracic disease or the same RT with concurrent continuous infusion of 1 g $m^{-2}$ $day^{-1}$ fluorouracil for 5 days. Survival, response and rates of symptom relief in the two groups were compared according to treatment intent, and toxicities were compared according to treatment received. The overall response rate was higher in patients randomized to the combination (29%) than in patients randomized to RT alone (16%) ($P = 0.035$). However, there were no significant differences between the treatment arms in terms of overall or progression-free survival or in palliation of symptoms. Patients treated with RT plus fluorouracil had significantly more acute toxicity, including nausea and vomiting ($P = 0.01$), oesophagitis ($P = 0.0003$), stomatitis ($P = 0.0005$) and skin reaction ($P = 0.003$). This study suggests for the first time an interaction between RT and infusional fluorouracil in NSCLC. Although RT plus fluorouracil resulted in a significantly higher response rate than achieved with RT alone, this did not translate into more effective palliation. Because the combination produced significantly more toxicity than RT alone, it is not recommended for the palliative treatment of NSCLC. Nevertheless, these results suggest that opportunities may exist for exploitation of the observed enhancement of anti-tumour effect in the setting of high-dose radical RT for NSCLC.

**Keywords:** lung cancer; radiotherapy; fluorouracil; palliation

Radiotherapy (RT) is an effective means of palliating thoracic symptoms in patients with incurable non-small-cell lung cancer (NSCLC) (Slawson and Scott, 1979). Cough, haemoptysis and chest pain were relieved in up to 65%, 75% and 85% of patients respectively in one randomized trial (Medical Research Council Lung Cancer Working Party, 1991)

It has been assumed that the degree of reduction in tumour size may correlate with the level of symptom relief and, since one previous study (Perez et al, 1982) has shown an RT dose–response relationship in NSCLC, the choice of palliative RT regimen usually represents a compromise between an adequate dose and an acceptable length of course of treatment. One strategy by which the biologically effective dose might be increased without extending overall treatment time may be to give RT concurrently with a radiosensitizing drug.

Fluorouracil has been known to enhance radiation-induced cell killing in vitro for over 30 years (Heidelberger et al, 1958). The requirements for maximum sensitization by fluorouracil of human tumour cells were studied by Byfield et al (1982). They observed that enhancement of cytotoxicity was strongly dependent on both the concentration of fluorouracil and the duration of exposure, and that only post-radiation exposure to the drug had a sensitizing (as opposed to additive) effect. The optimal conditions could only be achieved if the fluorouracil were administered by continuous infusion.

Continuous-infusion fluorouracil has been given concurrently with RT in a variety of tumours, including head and neck (Browman et al, 1994), oesophageal (Herskovic et al, 1992), rectal (O'Connell et al, 1994) and anal cancers (Cummings et al, 1991). The results of these studies have suggested that combined treatment is more effective than radiotherapy used alone. There has been only one previously reported randomized trial in which RT has been compared with RT plus fluorouracil in NSCLC (Carr et al, 1972). That study failed to reveal any evidence of an interaction between drug and radiation in terms of either response or effect on survival; however, the fluorouracil had been administered by bolus injection, which may be less effective than continuous infusion (O'Connell et al, 1994). Although there have been previous reports of fluorouracil given by infusion in conjunction with RT for NSCLC, these studies were not randomized (Kelly et al, 1989; Lokich et al, 1989).

The optimum dose and schedule of fluorouracil for concurrent use with RT for NSCLC are not known. The short half-life (< 15 min) of fluorouracil means that prolonged exposure before and after fractions of RT can best be achieved with a continuous infusion. Such an approach was shown in rectal carcinoma to be superior to bolus administration (O'Connell et al, 1994).

This study was designed as a randomized trial, which allowed us to test two hypotheses: first, that combined RT and infusional fluorouracil might increase response rates in NSCLC in comparison

*Received 13 May 1996*
*Revised 14 October 1996*
*Accepted 17 October 1996*

*Correspondence to:* D Ball with RT alone and, secondly, that increased response rates might result in longer duration and higher rates of palliation without increasing overall treatment time.

### PATIENTS AND METHODS

### Eligibility criteria

To be eligible for randomization, patients had to have histologically or cytologically proven NSCLC; disease unsuitable for either attempted curative resection or radical RT, or recurrent intrathoracic cancer outside any previously irradiated volume; measurable or evaluable disease; World Health Organization (WHO) performance status grade 0–3 (Miller et al, 1981) with life expectancy of at least 2 months; adequate bone marrow function with pretreatment haemoglobin $\geq$ 100 g $l^{-1}$, white cell count $\geq 3 \times 10^9$ $l^{-1}$ and platelet count $\geq 100 \times 10^9$ $l^{-1}$; serum creatinine < 0.15 mmol $l^{-1}$; adequate liver function with aspartate transaminase less than twice the upper limit of normal range; and no other serious medical or psychiatric illness. Before randomization, all patients had to give written informed consent and the protocol had been approved by the institutional ethics committees of the participating hospitals.

### Trial design

The trial was designed as a prospective non-blinded randomized trial to be conducted at Peter MacCallum Cancer Institute (PMCI). After the trial had been accruing patients for 3 years, the Royal Adelaide Hospital (RAH) became involved. Eligible patients were randomized to receive either RT alone or RT with concurrent fluorouracil with approximately equal probability. Central randomization was carried out by the Statistical Centre at PMCI with stratification for each participating hospital. A permuted block design was used for randomization at PMCI and a newer adaptive biased coin design for RAH.

It was originally planned to accrue 300 patients. This sample size was designed to have a 90% probability of detecting an increase in local response rate from 40% to 60% or a 50% increase in the median time to local failure with a two-tailed test at significance level 0.05. Planned interim analyses were carried out after accruing 100 and 200 patients respectively. Following the second interim analysis, the trial was closed because the acute toxic effects on the RT plus fluorouracil arm were significantly worse than on the RT arm, and there was no corresponding significant benefit in terms of palliation of symptoms or survival.

### Treatment methods

Before treatment, patients were simulated and the films marked so that the field covered the primary tumour with a 2-cm margin and the adjacent mediastinum. Anterior and posterior fields were treated daily to a total midplane dose of 20 Gy in five fractions. The dose and fractionation schedule were based on a radiotherapy regimen shown to be effective in the palliation of brain (Gelber et al, 1981) and bone metastases (Tong et al, 1982). Patients randomized to receive chemotherapy were given a continuous infusion of 1 g $m^{-2}$ $day^{-1}$ fluorouracil for 5 days, commencing as soon as possible on the same day after the first fraction of radiation (usually within 6 h) and concluding within 24 of the final increment. Prophylactic antiemetics were not routinely given to patients randomized to either arm.

### Patient assessment

Patients were assessed for treatment-related toxicity during and 1 month after treatment, then at each subsequent visit until acute toxic effects resolved. Toxicities were graded according to WHO criteria (Miller et al, 1981). For oesophagitis, for which there are no WHO criteria, the grades used were: 1, soreness; no medication; 2, soreness, requests medication; 3, soreness, liquid diet only; 4, alimentation not possible.

Criteria used for response and progression were those recommended by the WHO (Miller et al, 1981). Tumour size was measured in two dimensions by a single observer (the treating radiation oncologist) on pre- and post-treatment plain radiographs of the chest. If a complete or partial response was based on only one assessment, it was accepted provided there was no evidence of relapse within 4 weeks of achieving the response.

Although the presence of symptoms resulting from thoracic disease was not a criterion for eligibility, the majority of patients had one or more of four major symptoms, namely chest pain (42% of all patients), cough (82%), haemoptysis (35%) and dyspnoea (71%). These four symptoms were assessed before treatment and at each follow-up visit up to and including the date of progression of the treated tumour. The symptoms were graded by the clinician according to a five-point scale from absent (0) to very severe (4). Performance status (WHO) and weight loss were also documented before treatment and on each review.

Patients were asked to mark a set of seven linear analogue scales designed to assess their quality of life both before treatment and at each review up until progression of the treated tumour. Each linear analogue scale was 100 mm long with no divisions marked. Words representing the extremes of possible responses were written at the ends of each scale with the worst possible quality of life at the left hand end (0 mm). The questions asked were: in general how well do you feel? (extremes: very poorly, very well); how is your mood? (very miserable, very happy); how anxious do you feel? (very anxious, very relaxed); how well do you sleep? (very poorly, very well); how good is your appetite? (very poor, very good); how limited are you in your daily activities? (totally limited, not limited at all); and, if you compare the benefits of treatment with the side-effects, how worthwhile was the treatment? (not worthwhile at all, very worthwhile).

### Statistical methods

All analyses were carried out according to the randomized treatment arm. Acute toxic effects were also analysed according to the treatment actually given, and they have been presented in this paper, as the results were similar to those from the analysis by the randomized arm. The Wilcoxon rank sum test (two-tailed) was used to compare the worst grade of acute toxic effects in the two treatment groups, omitting patients who were not treated or whose toxic effects were not recorded.

The response rates were calculated as percentages of all randomized patients with 95 per cent confidence intervals (95% CI) estimated using the exact probabilities of the binomial distribution. The Yates corrected chi-square test or the Fisher exact test were used to compare response rates in the two arms.

All patients were followed to 14 February 1994, so this date was chosen as the close-out date for the analysis, i.e. all subsequent relapses and deaths were ignored. Only 12 patients were still alive on this date.

© Cancer Research Campaign 1997

Overall survival and progression-free survival curves were measured from the date of randomization and calculated using the Kaplan–Meier product-limit method. All causes of death were counted and patients were censored at the close-out date if they were still alive (overall survival curve) or alive without progression (progression-free survival curve). Ninety-five per cent confidence intervals for median survival or progression-free survival were estimated using the Brookmeyer–Crowley method and the Mantel–Cox log-rank test was used to compare patients according to randomized treatment arm. Multivariate analysis was carried out using the Cox proportional hazards model and the likelihood ratio test was used to assess the significance of the treatment arm in the model.

The following methods were used to compare the palliative effects of the two treatments:

1 Using linear interpolation between assessments, the average grade of each symptom was calculated from the date of randomization until the date of the last symptom assessment on or before progression of the treated tumour or, if there had been no progression, death or the close-out date. The average grade minus the grade at randomization was also calculated. Data were available for 89% of patients on the RT treatment arm and 88% on the RT plus fluorouracil arm. Comparison between treatment arms as carried out using the Wilcoxon rank sum test, two-tailed. Many patients did not initially have some of the symptoms being assessed, but they were included in the comparisons because they had the potential to develop them.
2 The estimated grades on each day were averaged over all patients still being assessed beyond that day and plotted separately by randomization arm for each symptom for the first year following randomization.
3 From the graphs obtained in (2), it was apparent that the maximum palliation of symptoms was achieved approximately 8 weeks after randomization. For each patient still being assessed, the grade of each symptom 8 weeks after randomization was subtracted from the initial grade and the differences compared according to treatment arm using the Wilcoxon rank sum test, two-tailed, omitting patients whose grades were unknown. Data were available for 64–67% of patients on the RT treatment arm and 77–78% on the RT plus fluorouracil arm, depending on symptom. This analysis was also done excluding those patients whose initial symptom grade was zero.

The quality of life data for the first six questions were analysed in a similar manner to the symptom data [methods (1) and (2)]. Data were available for 78% of patients on the RT treatment arm and 83% on the RT plus fluorouracil arm. For the seventh question (how worthwhile was the treatment) the response of each patient at 1,2,3,6 and 12 months following completion of treatment was obtained by interpolating between responses before and after each time point. The values obtained were averaged over all patients in each randomized arm who were still being assessed at that time. The number of patients available for analysis at each time point varied with the maximum being 53% of randomized patients 2 months after treatment.

BMDP Statistical Software was used in the analysis (Dixon, 1992).

**Table 1** Patient and disease characteristics according to randomized arm

| | RT ($n$ = 101) | RT + 5-FU ($n$ = 99) |
|---|---|---|
| Age at randomization (years) | | |
| Median | 67 | 67 |
| Range | 40–91 | 42–86 |
| | Percentage of patients | |
| Sex | | |
| Male:female | 70:30 | 79:21 |
| Histology | | |
| Squamous | 49 | 61 |
| Non-squamous | 49 | 38 |
| Mixed/unspecified | 3 | 1 |
| WHO performance status | | |
| 0 | 1 | 2 |
| 1 | 51 | 47 |
| 2 | 41 | 40 |
| 3 | 7 | 7 |
| Unknown | 0 | 3 |
| Weight loss in past 3 months | | |
| No | 40 | 35 |
| Yes | 60 | 63 |
| Unknown | 0 | 2 |
| Disease outside the irradiated area | | |
| None | 49 | 56 |
| Within chest | 16 | 11 |
| Outside chest | 36 | 33 |
| Prior treatment | | |
| None | 91 | 89 |
| Laser bronchoscopy | 4 | 4 |
| Surgery | 5 | 5 |
| Radiation to other sites ± surgery | 0 | 2 |
| Pretreatment chest pain | | |
| None | 54 | 59 |
| Mild | 27 | 20 |
| Moderate | 15 | 13 |
| Severe | 3 | 5 |
| Unknown | 1 | 3 |
| Pretreatment cough | | |
| None | 16 | 16 |
| Mild | 44 | 42 |
| Moderate | 32 | 32 |
| Severe | 8 | 5 |
| Very severe | 1 | 0 |
| Unknown | 0 | 4 |
| Pretreatment haemoptysis | | |
| None | 59 | 68 |
| Mild | 31 | 25 |
| Moderate | 9 | 4 |
| Severe | 1 | 0 |
| Unknown | 0 | 3 |
| Pretreatment dyspnoea | | |
| None | 28 | 27 |
| Mild | 28 | 23 |
| Moderate | 31 | 39 |
| Severe | 13 | 7 |
| Very severe | 1 | 0 |
| Unknown | 0 | 3 |

### RESULTS

### Patient population

Between August 1988 and July 1993, 204 patients were randomized but four were subsequently excluded because review revealed they had not met the eligibility criteria at randomization. The reasons for exclusion were small-cell carcinoma on pathology review (one patient), leiomyosarcoma at autopsy (one), oesophageal primary on review of radiology (one) and renal failure with incorrectly recorded serum creatinine (one). This left 200 patients eligible for analysis. Of these, 96% were treated at the Peter MacCallum Cancer Institute and 4% at the Royal Adelaide Hospital.

© Cancer Research Campaign 1997

**Table 2** Acute toxic effects according to treatment given

| | | RT (*n* = 106) | | RT + 5-FU (*n* = 90) | |
|---|---|---|---|---|---|
| | **Worst grade** | **No.** | **%[a]** | **No.** | **%[a]** |
| Nausea and vomiting | 0 | 57 | 58 | 35 | 39 |
| | 1 | 24 | 24 | 28 | 31 |
| | 2 | 15 | 15 | 19 | 21 |
| | 3 | 2 | 2 | 6 | 7 |
| | 4 | 1 | 1 | 1 | 1 |
| | Unknown | 7 | | 1 | |
| Oesophagitis | 0 | 59 | 60 | 31 | 35 |
| | 1 | 23 | 23 | 27 | 30 |
| | 2 | 14 | 14 | 20 | 22 |
| | 3 | 3 | 3 | 11 | 12 |
| | Unknown | 7 | | 1 | |
| Skin reaction | 0 | 83 | 86 | 60 | 68 |
| | 1 | 13 | 14 | 27 | 31 |
| | 2 | 0 | 0 | 1 | 1 |
| | Unknown | 10 | | 2 | |
| Stomatitis | 0 | 95 | 96 | 71 | 80 |
| | 1 | 3 | 3 | 7 | 8 |
| | 2 | 1 | 1 | 9 | 10 |
| | 3 | 0 | 0 | 2 | 2 |
| | Unknown | 7 | | 1 | |

[a]Percentage of patients with known values.

**Figure 1** Overall survival by randomized treatment arm

**Figure 2** Progression-free survival by randomized treatment arm

**Table 3** Best response of treated tumour according to randomized arm

| | **RT** | **RT + 5FU** |
|---|---|---|
| Complete response | 3 | 1 |
| Partial response | 13 | 28 |
| Stable disease | 45 | 47 |
| Progressive disease | 21 | 9 |
| Not evaluable | 19 | 14 |
| Total | 101 | 99 |
| Reason not evaluable | | |
| Not treated | 0 | 4 |
| Early death (≤ 7 weeks after RT) | 10 | 4 |
| Non-evaluable radiograph (fibrosis, effusion) | 5 | 5 |
| Patient did not return or withdrew consent | 3 | 1 |
| No pretreatment radiograph for comparison | 1 | 0 |
| Total | 19 | 14 |

Table 1 lists the characteristics of eligible patients at randomization. Pretreatment characteristics were reasonably balanced between the two arms except for the histology distribution (49% squamous cell carcinomas on RT compared with 61% on RT plus fluorouracil, $P = 0.15$). Staging for the presence of distant metastases was not routinely done because of the palliative nature of the treatment. Nevertheless, it can be seen that approximately half of the patients in each arm were known to have metastatic disease, either within or outside the thorax.

### Treatment

Of 101 patients randomized to RT alone, 99 (98%) received treatment as planned and two had breaks in treatment (one of these ceased RT after 16 Gy). Of 99 patients randomized to RT plus fluorouracil, 81 (82%) received treatment as planned, four patients received no treatment at all, five patients were treated with RT alone, three received less than the full dose of RT or fluorouracil and six patients experienced 1-day breaks or delays to the planned schedule of administration of combined treatment. Because patients randomized to RT alone were more likely to start treatment in the middle of the week, the duration of treatment was 6–8 days rather than 5 days in 29% of patients, compared with 9% of patients randomized to RT plus fluorouracil, since no treatment was given on Sundays.

### Acute toxicity

For the toxicity analysis, patients have been grouped according to treatment received, and four patients who were not treated have been omitted. Hence, comparisons have been made between 106 patients given RT alone and 90 patients given RT plus fluorouracil.

There were no significant differences in haematological toxicity between the groups, and no instances of grade 2,3 or 4 neutropenia or thrombocytopenia were recorded. Patients treated with the combination had significantly more nausea and vomiting ($P = 0.010$), oesophagitis ($P = 0.0003$), skin reaction ($P = 0.003$) and stomatitis ($P = 0.0005$) than patients treated with RT alone (Table 2). No other toxic effects differed significantly between the two treatment groups ($P > 0.05$).

*© Cancer Research Campaign 1997*

**Table 4** Palliation of symptoms according to randomization arm

| | Average minus initial grade | RT ($n$ = 101) No. | RT ($n$ = 101) %[a] | RT + 5-FU ($n$ = 99) No. | RT + 5-FU ($n$ = 99) %[a] |
|---|---|---|---|---|---|
| Chest pain ($P$ = 0.48) | Improved | 19 | 21 | 22 | 25 |
| | No change | 63 | 70 | 57 | 66 |
| | Worse | 8 | 9 | 8 | 9 |
| | Unknown | 11 | | 12 | |
| Cough ($P$ = 0.48) | Improved | 34 | 38 | 37 | 43 |
| | No change | 46 | 51 | 43 | 49 |
| | Worse | 10 | 11 | 7 | 8 |
| | Unknown | 11 | | 12 | |
| Haemoptysis ($P$ = 0.24) | Improved | 27 | 30 | 25 | 29 |
| | No change | 60 | 67 | 61 | 70 |
| | Worse | 3 | 3 | 1 | 1 |
| | Unknown | 11 | | 12 | |
| Dyspnoea ($P$ = 0.25) | Improved | 24 | 27 | 28 | 33 |
| | No change | 56 | 62 | 48 | 56 |
| | Worse | 10 | 11 | 10 | 12 |
| | Unknown | 11 | | 13 | |

[a]Percentage of patients with known values. Improved, improvement of at least 0.5 grade compared with initial grade; worse, deterioration of more than 0.5 grade compared with initial grade; no change, neither better nor worse, including patients who never had the symptom. $P$-values are for comparisons between the randomization arms using the Wilcoxon rank sum test on actual average minus initial grades, i.e. not on the grouped data.

**Table 5** Palliation of symptoms according to randomization arm (omitting patients with grade 0 initially)

| | Average minus initial grade | No. | %[a] | No. | %[a] |
|---|---|---|---|---|---|
| | | RT ($n$ = 46) | | RT + 5-FU ($n$ = 41) | |
| Chest pain ($P$ = 0.28) | Improved | 19 | 51 | 22 | 65 |
| | No change | 16 | 43 | 10 | 29 |
| | Worse | 2 | 5 | 2 | 6 |
| | Unknown | 9 | | 7 | |
| | | RT (85) | | RT + 5-FU (83) | |
| Cough ($P$ = 0.48) | Improved | 34 | 45 | 37 | 51 |
| | No change | 39 | 51 | 32 | 44 |
| | Worse | 3 | 4 | 4 | 5 |
| | Unknown | 9 | | 10 | |
| | | RT (41) | | RT + 5-FU (32) | |
| Haemoptysis ($P$ = 0.61) | Improved | 27 | 73 | 25 | 93 |
| | No change | 9 | 24 | 2 | 7 |
| | Worse | 1 | 3 | 0 | 0 |
| | Unknown | 4 | | 5 | |
| | | RT (73) | | RT + 5-FU (72) | |
| Dyspnoea ($P$ = 0.21) | Improved | 24 | 37 | 28 | 45 |
| | No change | 37 | 57 | 30 | 48 |
| | Worse | 4 | 6 | 4 | 6 |
| | Unknown | 8 | | 10 | |

[a]Percentage of patients with known values. Improved, improvement of at least 0.5 grade compared with initial grade; worse, deterioration of more than 0.5 grade compared with initial grade; no change, neither better nor worse. $P$-values are for comparisons between the randomization arms using the Wilcoxon rank sum test on actual average minus initial grades, i.e. not on the grouped data.

Other instances of grade 3 or 4 toxicity included diarrhoea in one patient given RT plus fluorouracil, pulmonary toxicity in two patients given RT alone, spinal cord injury in one patient given RT plus fluorouracil and fatigue/lethargy in one patient each given RT alone and RT plus fluorouracil, but for these toxicities there were no significant differences between the groups. One patient treated with the combination developed moderate angina requiring discontinuation of fluorouracil.

### Response and survival

The best response of the treated tumour, as measured from plain radiographs, was recorded according to the intention to treat as in Table 3. The overall response rate for patients randomized to the combination was 29% (95% CI 21–39%), which was significantly higher than the response rate of 16% (95% CI 9–24%) for patients randomized to RT alone ($P$ = 0.035).

© Cancer Research Campaign 1997

Number being followed

| | | | | | | | | | | | | | | |
|---|---|---|---|---|---|---|---|---|---|---|---|---|---|---|
| Grade 3 | 1 | 0 | | | | | | | | | | | | |
| Grade 2 | 12 | 12 | 10 | 5 | 4 | 4 | 3 | 2 | 2 | 1 | 1 | 1 | 1 | 0 |
| Grade 1 | 51 | 51 | 45 | 39 | 31 | 23 | 21 | 17 | 11 | 9 | 8 | 7 | 4 | 4 |
| Grade 0 | 113 | 109 | 90 | 70 | 54 | 51 | 39 | 32 | 24 | 18 | 17 | 12 | 11 | 11 |

**Figure 3** Haemoptysis by initial grade. Higher grade = worse haemoptysis

Number being followed

| | | | | | | | | | | | | | | |
|---|---|---|---|---|---|---|---|---|---|---|---|---|---|---|
| RT | 90 | 86 | 68 | 51 | 39 | 36 | 28 | 23 | 18 | 11 | 11 | 9 | 7 | 7 |
| RT + 5-FU | 87 | 86 | 77 | 63 | 50 | 42 | 35 | 28 | 19 | 17 | 15 | 11 | 9 | 8 |

**Figure 4** Haemoptysis by randomized treatment arm. Higher grade = worse haemoptysis

**Table 6** Quality of life according to randomization arm

| | | RT (*n* = 101) | | RT + 5-FU (*n* = 99) | |
|---|---|---|---|---|---|
| | **Average minus initial level** | **No.** | **%[a]** | **No.** | **%[a]** |
| In general, how well do you feel? (*P* = 0.47) | Improved | 23 | 29 | 20 | 24 |
| | No change | 35 | 44 | 35 | 43 |
| | Worse | 22 | 28 | 27 | 33 |
| | Unknown | 21 | | 17 | |
| How is your mood? (*P* = 0.51) | Improved | 24 | 30 | 21 | 26 |
| | No change | 36 | 46 | 41 | 50 |
| | Worse | 19 | 24 | 20 | 24 |
| | Unknown | 22 | | 17 | |
| How anxious do you feel? (*P* = 0.11) | Improved | 27 | 34 | 26 | 32 |
| | No change | 42 | 53 | 36 | 44 |
| | Worse | 10 | 13 | 20 | 24 |
| | Unknown | 22 | | 17 | |
| How well do you sleep? (*P* = 0.57) | Improved | 23 | 29 | 23 | 28 |
| | No change | 35 | 44 | 35 | 43 |
| | Worse | 21 | 27 | 24 | 29 |
| | Unknown | 22 | | 17 | |
| How good is your appetite? (*P* = 0.39) | Improved | 27 | 34 | 23 | 28 |
| | No change | 32 | 41 | 32 | 39 |
| | Worse | 20 | 25 | 27 | 33 |
| | Unknown | 22 | | 17 | |
| How limited are you in your daily activities? (*P* = 0.30) | Improved | 23 | 29 | 15 | 18 |
| | No change | 35 | 44 | 40 | 49 |
| | Worse | 21 | 27 | 27 | 33 |
| | Unknown | 22 | | 17 | |

[a]Percentage of patients with known values. Improved, improvement of at least 10 mm compared with initial level; worse, deterioration of more than 10 mm compared with initial level; no change, neither better nor worse. *P*-values are for comparisons between the randomization arms using the Wilcoxon rank sum test on actual average minus initial levels, i.e. not on the grouped data.

By the close-out date, 92% of patients randomized to RT and 96% of patients randomized to RT plus fluorouracil had died. Survival measured from the date of randomization for all 200 patients according to treatment intent is shown in Figure 1. The estimated median survival for patients randomized to RT was 6.0 months (95% CI 5.0–7.9 months) with an estimated 26% alive at 1 year and 4% at 2 years. The estimated median survival for patients randomized to RT plus fluorouracil was 6.8 months (95% CI 5.8–8.0 months) with an estimated 26% alive at 1 year and 9% at 2 years. There was no significant difference in survival between the two treatment arms (*P* = 0.36).

Multivariate analysis, adjusting for potential prognostic factors, namely pretreatment performance status grade 2 or 3, weight loss, non-squamous histology and presence of disease outside the irradiated area, confirmed that the randomized treatment arm had no significant influence on survival (*P* = 0.33).

Progression-free survival is shown in Figure 2. The estimated median progression-free survival for patients randomized to RT was 3.0 months (95% CI 2.3–3.4 months) with an estimated 6% alive without progression at 1 year. The estimated median progression-free survival for patients randomized to RT plus fluorouracil was 3.8 months (95% CI 3.2–4.2 months) with an estimated 9% alive without progression at 1 year. The difference between arms was not statistically significant ($P = 0.073$). This result was confirmed after adjustment for the four potential prognostic factors in a multivariate model ($P = 0.086$).

### Palliation of symptoms

For each of the four symptoms, there were no significant differences between the two randomization arms in initial grade ($P \geq 0.09$), average grade following randomization ($P \geq 0.17$), average minus initial grades ($P \geq 0.24$) or 8-week minus initial grades ($P \geq 0.13$). Table 4 shows the average minus initial grades according to randomization arm. Table 5 shows the same results after excluding patients who did not have the symptom initially.

Figure 3 shows the average grade of haemoptysis plotted over time according to initial grade, and Figure 4 shows the average grade according to randomized treatment arm. Similar plots (not shown) were prepared for the other three symptoms assessed.

The average grade of symptoms was lower after randomization for 45 patients who achieved complete or partial response compared with 122 patients who had stable or progressive disease (chest pain $P = 0.022$, cough $P = 0.0006$, haemoptysis $P = 0.055$, and dyspnoea $P = 0.0098$). However, when the initial symptom grade was subtracted from the average grade post-randomization, there were no significant differences between responders and non-responders ($P \geq 0.065$).

### Quality of life

Table 6 shows the changes in responses to each of the six quality of life questions. There were no significant differences between randomization arms in the average minus initial levels recorded for any of these questions ($P \geq 0.11$). There were no significant differences between arms with respect to whether the patients considered their treatment to be worthwhile at any of the five specified times following completion of treatment ($P \geq 0.15$).

### DISCUSSION

One previous randomized comparison of RT *vs* combined RT and bolus fluorouracil in NSCLC failed to reveal any evidence of an interaction between drug and radiation either in terms of response or effect on survival (Carr et al, 1972). Since then, the feasibility of delivering fluorouracil by continuous infusion concurrently with both palliative (Kelly et al, 1989) and radical RT (Lokich et al, 1989) for NSCLC has been established but, because these were both single-arm studies, no conclusions were possible with regard to the effects of combined treatment on response rates, survival and toxicity.

In the current study, combined treatment using infusional fluorouracil increased the local response rate. There was also a non-significant increase in the duration of progression-free survival with the combined treatment. Our results thus suggest, for the first time, an interaction between RT and infusional fluorouracil in NSCLC but, because the trial did not contain a chemotherapy-only arm, we are unable to say whether the interaction is additive or synergistic. The limited information available on the response of NSCLC to infusional fluorouracil as a single agent suggests that it has low activity – 8% response rate in one phase II study (Citron et al, 1992). In our trial, the difference in response between treatment arms was 13%.

We also observed an increase in acute toxicities associated with the combination compared with RT alone. It is possible that a similar outcome (increased response rates and increased toxicities) may have been achieved simply by increasing the total radiation dose, in which case there has been no therapeutic gain. The acute toxicities were tolerable (grades 1–2) in the majority of patients, but of particular concern was the development of spinal cord injury in one patient shortly after the completion of combination treatment. The clinical features and magnetic resonance imaging (MRI) scan appearances were consistent with radiation myelopathy; however, such an isolated event suggests that the patient may have been genetically susceptible to radiation injury and any role of fluorouracil in the development of this complication remains speculative.

This study has confirmed that RT is an effective means of relieving symptoms, especially chest pain and haemoptysis, in patients with advanced NSCLC. Unlike previous studies of palliative RT in NSCLC, we have included in the analyses those patients who did not have the symptom initially. A few of these patients subsequently went on to develop new symptoms in spite of RT, and we felt that account should be taken of what are effectively treatment failures. Using this method, the rates of palliation appear inferior to those reported in the British Medical Research Council (MRC) studies, but when only those patients with the symptom present initially are analysed (Table 5), the rates of symptom relief in this and the MRC studies are similar. For example, cough, haemoptysis and chest pain improved in 48%, 81% and 58%, respectively, of patients in this study compared with 52%, 73% and 66% of patients with corresponding symptoms in one of the MRC trials (Medical Research Council Lung Cancer Working Party, 1992).

The relationship, if any, between objective response rate and palliation success in NSCLC is unclear. Although a dose–objective response relationship was evident in a RTOG study of definitive radical RT (Perez et al, 1982), a similar relationship was not found in a palliative study conducted by the same group (Simpson et al, 1985), nor was increasing dose associated with higher rates of symptom relief. None of the three MRC studies (Medical Research Council Lung Cancer Working Party, 1991, 1992; British Medical Research Council Lung Cancer Working Party, 1994) of palliative RT has shown differences in palliation between several different fractionation regimens, although higher doses were associated with more severe oesophagitis in two of the studies (Medical Research Council Lung Cancer Working Party, 1992; British Medical Research Council Lung Cancer Working Party, 1994). and with longer survival in one (British Medical Research Council Lung Cancer Working Party, 1994). In a recently reported randomized trial from South Africa, a higher-dose regimen was not associated with higher symptom or objective tumour response, although it did produce more severe oesophagitis (Abratt et al, 1995). Our study is in keeping with these observations in that the addition of infusional fluorouracil, although increasing the response rate, did not result in significantly better palliation or quality of life. The reasons for this are unclear, but one could speculate that current techniques for assessing palliation success are

© Cancer Research Campaign 1997

not sufficiently refined to detect treatment benefits. Alternatively, the doses used in the palliative range for NSCLC may lie below the threshold above which any sigmoid dose–response relationship for symptom relief becomes evident. Absence of a clear dose–response relationship has been consistently observed in other palliative situations, including the treatment of bone (Tong et al, 1982) and brain metastases (Gelber et al, 1981).

Whatever benefits may have resulted from the use of the combination, e.g. slightly longer progression-free survival in patients with limited disease, have been at the cost of greater toxicity and, although the toxicity was relatively mild, we are unable to recommend the combination regimen as palliation for NSCLC. The observed but previously unreported interaction between fluorouracil and radiation in NSCLC may nevertheless provide opportunities for further study in patients receiving radical RT for NSCLC.

### ACKNOWLEDGMENTS

We wish to thank the data managers who collected the data and helped in the organization of the trial at each institution, namely Jill Dipell, Janey Stone and Ann-Maree Hayes at the Peter MacCallum Cancer Institute, and Nancy Olszewski at the Royal Adelaide Hospital. This trial was supported by a research grant from the National Health and Medical Research Council of Australia.

### REFERENCES

Abratt RP, Shepherd LJ and Mameena Salton DG (1995) Palliative radiation for stage 3 non small cell lung cancer: a prospective study of two moderately high dose regimens. *Lung Cancer* **13:** 137–143

British Medical Research Council Lung Cancer Working Party (1994) Randomised trial of two radiotherapy (RT) policies for patients with inoperable non-small cell lung cancer (NSCLC) and good performance status. *Lung Cancer* **11** (Suppl. 1): 131

Browman GP, Cripps C, Hodson DI, Eapen L, Sathya J and Levine MN (1994) Placebo-controlled randomized trial of infusional fluorouracil during standard radiotherapy in locally advanced head and neck cancer. *J Clin Oncol* **12:** 2648–2653

Byfield JE, Calabro-Jones P, Klisak I and Kulhanian F (1982) Pharmacologic requirements for obtaining sensitization of human tumor cells *in vitro* to combined 5-fluorouracil or ftorafur and x rays. *Int J Radiat Oncol Biol Phys* **8:** 1923–1933

Carr DT, Childs DS and Lee RE (1972) Radiotherapy plus 5-FU compared to radiotherapy alone for inoperable and unresectable bronchogenic carcinoma. *Cancer* **29:** 375–380

Citron ML, Modeas C, Propert K, Goutsou M and Green MR (1992) Phase II trial of high-dose 24-hour continuous intravenous 5-fluorouracil for advanced non-small cell lung cancer: a Cancer and Leukemia Group B study. *Cancer Invest* **10:** 215–219

Cummings BJ, Keane TJ, O'Sullivan B, Wong CS and Catton CN (1991) Epidermoid anal cancer: treatment by radiation alone or by radiation and 5-fluorouracil with and without mitomycin C. *Int J Radiat Oncol Biol Phys* **21:** 1115–1125

Dixon WJ (ed) (1992) *BMDP Statistical Software Manual*. University of California Press: Berkeley

Gelber RD, Larson M, Borgelt BB and Kramer S (1981) Equivalence of radiation schedules for the palliative treatment of brain metastases in patients with favorable prognosis. *Cancer* **48:** 1749–1753

Heidelberger C, Griesbach L, Montag BJ, Mooren D, Cruz O, Schnitzer RJ and Grunberg E (1958) Studies on fluorinated pyrimidines. II. Effects on transplanted tumors. *Cancer Res* **18:** 305–317

Herskovic A, Martz K, Al-Sarraf M, Leichman L, Brindle J, Vaitkevicius V, Cooper J, Byhardt R, Davis L and Emami B (1992) Combined chemotherapy and radiotherapy compared with radiotherapy alone in patients with carcinoma of the esophagus. *N Engl J Med* **326:** 1593–1598

Kelly SA, Macleod PM and Ash DV (1989) The use of simultaneous radiotherapy and 5-fluorouracil in patients with inoperable squamous cell lung cancer. *Clin Radiol* **40:** 311–313

Lokich J, Chaffey J and Neptune W (1989) Concomitant 5-fluorouracil infusion and high dose radiation for stage III non-small cell lung cancer. *Cancer* **64:** 1021–1025

Medical Research Council Lung Cancer Working Party (1991) Inoperable non-small-cell lung cancer (NSCLC): a Medical Research Council randomised trial of palliative radiotherapy with two fractions or ten fractions. *Br J Cancer* **63:** 265–270

Medical Research Council Lung Cancer Working Party (1992) A Medical Research Council (MRC) randomised trial of palliative radiotherapy with two fractions or a single fraction in patients with inoperable non-small-cell lung cancer (NSCLC) and poor performance status. *Br J Cancer* **65:** 934–941

Miller AB, Hoogstraten B, Staquet M and Winkler A (1981) Reporting results of cancer treatment. *Cancer* **47:** 207–214

O'Connell MJ, Martenson JA, Wieand HS, Krook JE, Macdonald JS, Haller DG, Mayer RJ, Gunderson LL and Rich TA (1994) Improving adjuvant therapy for rectal cancer by combining protracted-infusion fluorouracil with radiation therapy after curative surgery. *N Engl J Med* **331:** 502–507

Perez CA, Stanley K, Grundy G, Hanson W, Rubin P, Kramer S, Brady LW, Marks JE, Perez-Tamayo R, Brown GS, Concannon JP and Rotman M (1982) Impact of irradiation technique and tumor extent in tumor control and survival of patients with unresectable non-oat cell carcinoma of the lung. *Cancer* **50:** 1091–1099

Simpson JR, Francis ME, Perez-Tamayo R, Marks RD and Rao DV (1985) Palliative radiotherapy for inoperable carcinoma of the lung: final report of a RTOG multi-institutional trial. *Int J Radiat Oncol Biol Phys* **11:** 751–758

Slawson RG and Scott RM (1979) Radiation therapy in bronchogenic carcinoma. *Radiology* **132:** 175–176

Tong D, Gillick L and Hendrickson FR (1982) The palliation of symptomatic osseous metastases. *Cancer* **50:** 893–899

© Cancer Research Campaign 1997